# Synthetic European road freight transport flow data

**DOI:** 10.1016/j.dib.2021.107786

**Published:** 2022-01-02

**Authors:** Daniel Speth, Verena Sauter, Patrick Plötz, Tim Signer

**Affiliations:** a Fraunhofer Institute for Systems and Innovation Research ISI, Breslauer Str. 48, Karlsruhe 76139, Germany; b Karlsruhe Institute for Technology (KIT), Institute for Industrial Production (IIP), Hertzstr. 12, Karlsruhe 76185, Germany

**Keywords:** Transport, Road freight transport, Truck-transport, Origin-destination, Traffic, Traffic flow

## Abstract

This data article describes a dataset on European road freight traffic. The dataset includes truck traffic flows between 1675 regions all over Europe. In addition to the road freight flows in tons as well as number of vehicles, the dataset also contains the shortest path between the respective regions on the European highway network (E-roads). Fifteen columns provide the following information for each pair of regions: (1) ID origin region, (2) name origin region, (3) ID destination region, (4) name destination region, (5) path in the E-road network, (6) distance from origin region to the E-road network, (7) distance within the E-road network, (8) distance from the E-road network to the destination region, (9) total distance, (10) road freight flow in tons for 2010, (11) road freight flow in tons for 2019, (12) road freight flow in tons for 2030, (13) truck traffic flow in number of vehicles for 2010, (14) truck traffic flow in number of vehicles for 2019, (15) truck traffic flow in number of vehicles for 2030. In addition, a table of nodes and a table of edges of the modelled E-road network is available. Finally, a list with supplementary information on the regions under consideration is given. In 2010, the ETISplus project collected Europe-wide freight volumes from various EU sources as well as from the EU countries and calibrated the resulting origin-destination matrices with measured traffic flows. For the dataset described here, the road freight volume was updated using Eurostat data and a forecast up to 2030 was added. The freight volume was converted into vehicles travelling. Subsequently, the highway network relevant for trucks was extracted from the ETISplus project and manually updated with the current E-road network. Finally, each origin-destination freight volume was allocated to the network using Dijkstra's algorithm. This provides a synthetically generated road freight traffic volume for each road section. The generated data provide an extremely relevant basis for the design of future road infrastructure in Europe, for example hydrogen refuelling stations or charging stations for electric trucks. Thus, the data are not only relevant for traffic science studies, but also of high importance for planners in practice.

**Specifications Table**

**Table:** 

Subject	Business, Management and decision sciences
Specific subject area	Transportation management; Future traffic infrastructure planning
Type of data	Table (.csv)
How data were acquired	Primary source: Publicly available data from the ETISplus project on truck traffic volumes in Europe 2010 [1]. Update: Publicly available Eurostat data [2] processed using Python code Converting freight volume to vehicles: Publicly available Eurostat data [3] processed using Python code Network calculations: Python library NetworkX [4], that contains an implementation of Dijkstra's algorithm For a detailed description see Section 2
Data format	Raw Data Analyzed Processed (data update, data conversion and data enrichment, network calculation)
Parameters for data collection	Data was collected exclusively from publicly available sources on truck traffic volumes. It covers European freight transport. The data was updated to 2019 and a forecast was performed for 2030.
Description of data collection	Data on road freight transport was taken from the original dataset of the ETISplus project [1]. Backward search was used to identify ETISplus input data. The identified Eurostat datasets [2,3], in their most current edition, are used to generate the secondary dataset described here.
Data source location	Primary data sources: • ETISplus general information [1] • ETISplus road freight matrix [5] • ETISplus nodes and edges from road network (land networks) [6] • ETISplus NUTS-3 regions [7] • Eurostat national and international transport data [2] • Eurostat empty runs of trucks [3]
Data accessibility	Repository name: Mendely Data identification number: 10.17632/py2zkrb65h.1 Direct URL to data: https://data.mendeley.com//datasets/py2zkrb65h/1

**Value of the Data**

• Heavy road freight transport in Europe is facing significant changes due to stricter climate targets. The dataset provides a unique basis for estimating future infrastructure needs, e.g. charging locations or hydrogen refuelling stations, across Europe. Thus, the dataset creates a basis for future policy decisions in road freight transport.
• The dataset is of interest for researchers and companies and has an influence on political decisions. Scientists can develop their own traffic models based on the data. Infrastructure providers can use the data as a basis for estimating market potential; energy suppliers can derive future demand from road freight transport. Policymakers can benefit from these considerations.
• The dataset can be used to estimate charging or refuelling infrastructure for trucks, for example by using Flow-Refuelling Location Models (FRLM).
• Traffic scientists can use the dataset to generate synthetic driving profiles for trucks and thus optimize routing.
• Energy system modellers can use the dataset to estimate future regional distribution of energy demand from road freight transport as a model input.

## 1 Data Description

The data publication consists of four individual datasets: (1) the truck traffic flow data, (2) an overview of the included NUTS-3 regions^1^, (3) a list of the network nodes in the underlying road network, (4) a list of the network edges in the underlying road network. The first dataset is the central one; the other datasets provide additional information. All datasets are available as comma-separated values (.csv) and are zipped for better storability. Commas separate columns; the dot is used as decimal separator. The datasets are available via Mendeley [9].

In the following, each individual dataset is briefly described. For each dataset, the variables used are listed in a table.

The first dataset *01_Trucktrafficflow* provides information about the updated traffic flows between each NUTS-3 region. In total, the dataset considers 1,514,573 directed transport flows between 1630 different origins (NUTS-3) and 1667 destinations (NUTS-3). The dataset contains the individual transport flow between a pair of regions as rows. The difference between origins and destinations arises because no outgoing transport flows are known for Monte Negro, Kazakhstan, and Gibraltar. The number of transport flows is smaller than the product of origin and destination, since not every region ships goods to every other region. In addition, transport flows within a region are not taken into account. The first four columns contain information on the origin and destination region of the respective transport flow under consideration. These nomenclature data are taken directly from [7]. The next column contains the route on the E-road network as a list of nodes passed from the origin to the destination. The network consists of all European international E-roads supplemented by other highways that are relevant for road freight transport. A description on the selection process can be found in Section 2. The routes are modelled as shown in Section 2. The following four columns contain information about the distance travelled in kilometres. First, the distance from the centre of the origin region to the E-road network is given as haversine distance. The next column contains the distance travelled on the E-road network using Dijkstra's algorithm. Afterwards, the distance from the E-road network to the centre of the destination region is given. Finally, the last column on distance provides the sum of all distance, i.e. the distance from the centre of the origin region to the centre of the destination region. Since the distances are based on the route, they are also modelled values. Subsequently, freight flows for 2010, 2019, and 2030 in tons are presented. The 2010 data are the values originally calculated in the ETISplus project [1] provided in the ETISplus database [5]. The figures for 2019 and 2030 are newly calculated values. Finally, the last three columns provide the calculated annual number of trucks, travelling the relevant route in 2010, 2019 and 2030. A short description of each variable in the truck traffic flow dataset can be found in Table 1. Table 2 presents an exemplary excerpt from the dataset for two transport flows. Please note that the modelling of traffic paths and the projected traffic flows are subject to simplifications, which are explained in Section 2.

**Table 1:** Description of variables used in the truck traffic flow dataset (01_Trucktrafficflow).

Name	Description	Data Type	Unit	Source
ID_origin_region	Unique record ID with 9 digits decoding NUTS-3 region of origin. First 3 digits decode NUTS-0, first 5 decode NUTS-1, first 7 decode NUTS-3	Integer (9digits)	-	Adapted from [7]
Name_origin_region	National name of NUTS-3 region of origin	String	-	Adapted from [7]
ID_destination_region	Unique record ID with 9 digits decoding NUTS-3 code of destination region. First 3 digits decode NUTS-0, first 5 decode NUTS-1, first 7 decode NUTS-3	Integer (9digits)	-	Adapted from [7]
Name_destination_ region	National name of NUTS-3 destination region	String	-	Adapted from [7]
Edge_path_E_road	List of the *network edge IDs* of the shortest path between the O-D pair, determined with Dijkstra's algorithm	String	-	Modelled
Distance_from_origin_ region_to_E_road	Distance from the geometric centre of the origin region to the closest network node	Float	Kilometres [km]	Modelled
Distance_within_E_ road	Distance of the shortest edge path between the O-D pair	Float	Kilometres [km]	Modelled
Distance_from_E_ road_to_destination_ region	Distance from the geometric centre of the destination region to the closest network node	Float	Kilometres [km]	Modelled
Total_distance	Sum of *Distance_from_origin_region_to_E_road, Distance_within_E_road* and *Distance_from_E_road_to_destination_region*	Float	Kilometres [km]	Modelled
Traffic_flow_trucks_ 2010	Number of trucks that drive between the O-D pair in 2010	Float	Number of trucks	Modelled
Traffic_flow_trucks_ 2019	Number of trucks that drive between the O-D pair after they had been scaled to 2019	Float	Number of trucks	Modelled
Traffic_flow_trucks_ 2030	Number of trucks that drive between the O-D pair according to the forecast for 2030	Float	Number of trucks	Modelled
Traffic_flow_tons_ 2010	Number of tons that are transported between the O-D pair in 2010 according to ETISplus	Integer	Tons [t]	Adapted from [5]
Traffic_flow_tons_ 2019	Number of tons that are transported between the O-D pair after they had been scaled to 2019	Integer	Tons [t]	Modelled
Traffic_flow_tons_ 2030	Number of tons that are transported between the O-D pair according to the forecast for 2030	Integer	Tons [t]	Modelled

**Table 2:** Exemplary extract of the truck traffic flow dataset (01_Trucktrafficflow).

**ID_origin_region**	**Name_origin_region**	**ID_destination_region**	**Name_destination_region**	**Edge_path_E_road**
101010101	Mittelburgenland	101010201	Mostviertel-Eisenwurzen	[1034974, 1008535, ..., 1008578]
101010101	Mittelburgenland	101010203	Sankt Polten	[1034974, 1008535, ..., 1008682]

**Distance_from_origin_** **region_to_E_road**	**Distance_within_** **E_road**	**Distance_from_E_road_to_** **destination_region**	**Total_distance**	**Traffic_flow_trucks_2010**

30	160	9	199	493.75
30	110	5	145	556.25

**Traffic_flow_** **trucks_2019**	**Traffic_flow_** **trucks_2030**	**Traffic_flow_** **tons_2010**	**Traffic_flow_** **tons_2019**	**Traffic_flow_** **tons_2030**

511.25	533.75	6715	6953	7259
576.25	601.25	7565	7837	8177

The second dataset *02_NUTS-3-Regions* contains information on the considered 1,675 NUTS-3 regions. The content was taken from the original ETISplus project file *_EZ_2006_3.csv* [7]. Data on the regions themselves (ID, name, country) and the location of their centres are included. In addition, we assigned the nearest point in the E-road network. Table 3 provides detailed information on the variables used.

**Table 3:** Description of variables used in the NUTS-3 regions dataset (02_NUTS-3-Regions), adapted from [7].

Name	Description	Data Type	Unit
ETISplus_Zone_ID	Unique record ID with 9 digits decoding NUTS-3 region. First 3 digits decode NUTS-0, first 5 decode NUTS-1, first 7 decode NUTS-3	Integer (9digits)	-
Name	National name of the NUTS-3 region	String	-
Country	Unique country code of the country in which the NUTS-3 region is located (country codes are defined by ETISplus)	String (2digits)	-
Geometric_centre	Defines the geometric centre of the NUTS-3 region	String	-
Geometric_centre_X	Longitude of the geometric centre of the NUTS-3 region	Float	Degrees
Geometric_centre_Y	Latitude of the geometric centre of the NUTS-3 region	Float	Degrees
Network_Node_ID	Unique ID of the network node with the shortest distance to the geometric centre of the origin region	Integer	-

The datasets *03_network-nodes* and *04_network_edges* describe the underlying E-road network. The network consists of 17,435 nodes and 18,447 edges, which are represented as individual lines in the datasets. Each node has a unique ID and unique coordinates. In addition, both the NUTS-3 region and the country in which the node is located is identified. Each edge also has a unique ID. An edge always connects exactly two nodes with each other. For each edge, it is also indicated whether it comes from the original ETISplus dataset [6] or was added manually. Finally, we provided truck traffic volumes on each edge for 2019 and 2030. Tables 4 and 5 provide all relevant information on the variables used to define the network.

**Table 4:** Description of variables used in the network nodes list (03_network-nodes).

Name	Description	Data Type	Unit	Source
Network_Node_ID	Unique network node ID	Integer (6 digits)	-	Adapted from [6]
Network_Node_X	Longitude of the location of network node	Float	Degrees	Adapted from [6]
Network_Node_Y	Latitude of the location of network node	Float	Degrees	Adapted from [6]
ETISplus_Zone_ID	ID of the NUTS-3 region in which the network node is located	Integer	-	Adapted from [6]
Country	Unique country code of the country in which the network node is located (country codes are defined by ETISplus)	String	-	Adapted from [6]

**Table 5:** Description of variables used in the network edges list (04_network-edges).

Name	Description	Data Type	Unit	Source
Network_Edge_ID	Unique edge ID	Integer (7 digits)	-	Adapted from [6]
Manually_Added	Determines whether an edge had been manually added to the network (1) or not (0)	Binary-integer	-	Modelled
Distance	Length of the network edge	Float	Kilometres [km]	Adapted from [6]
Network_Node_A_ID	Unique ID of the network node that defines one end point of the network edge	Integer	-	Adapted from [6]
Network_Node_B_ID	Unique ID of the network node that defines one end point of the network edge	Integer	-	Adapted from [6]
Traffic_flow_trucks_2019	Number of trucks that drive on the edge in 2019 (both highway directions combined)	Float	Number of trucks	Modelled
Traffic_flow_trucks_2030	Number of trucks that drive on the edge in 2030 (both highway directions combined)	Float	Number of trucks	Modelled

**Fig. 1:**
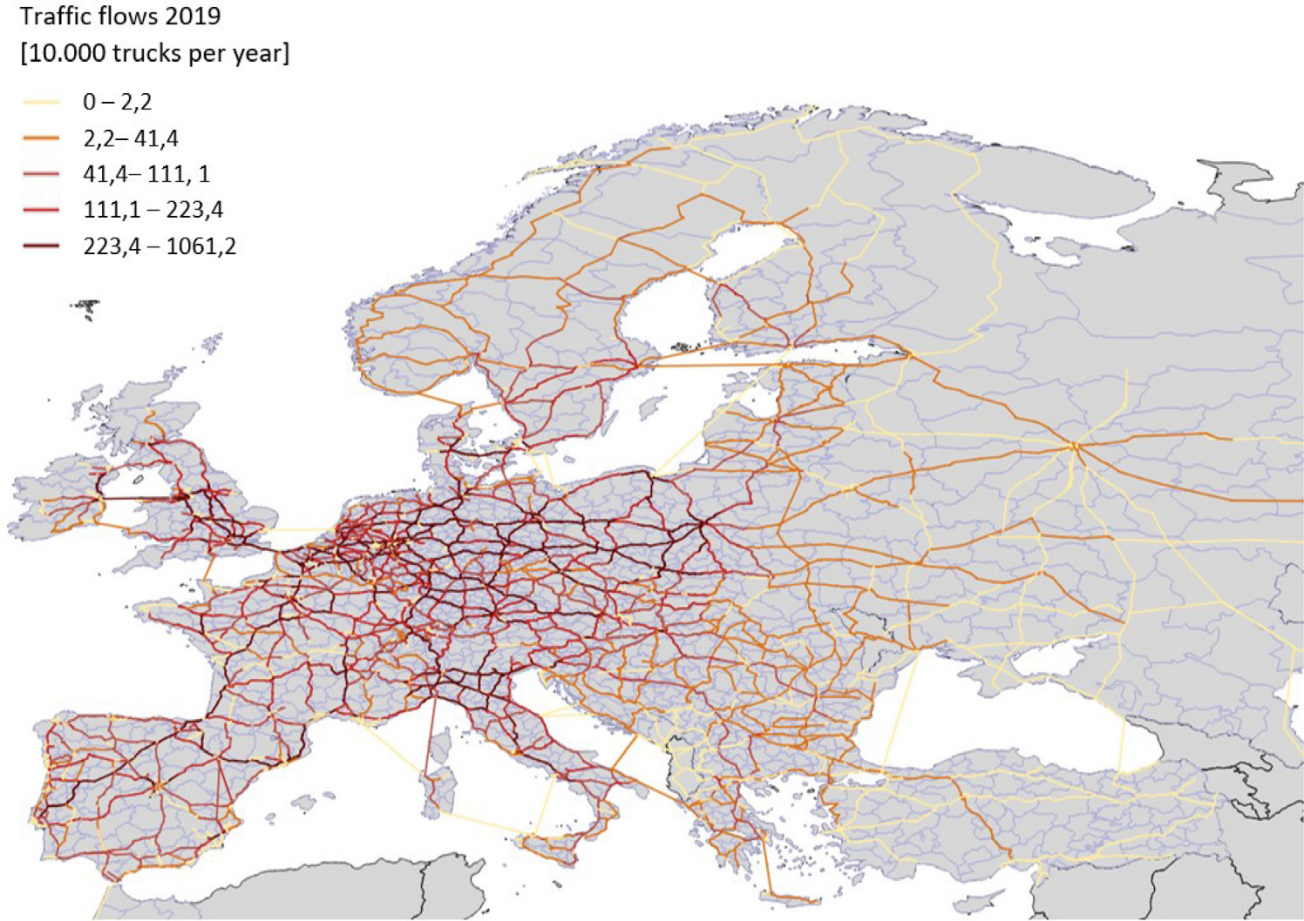
Modelled truck traffic flows in 2019.

Fig. 1 illustrates the modeled road network and the calculated traffic flows in 2019.

**Fig. 2:**
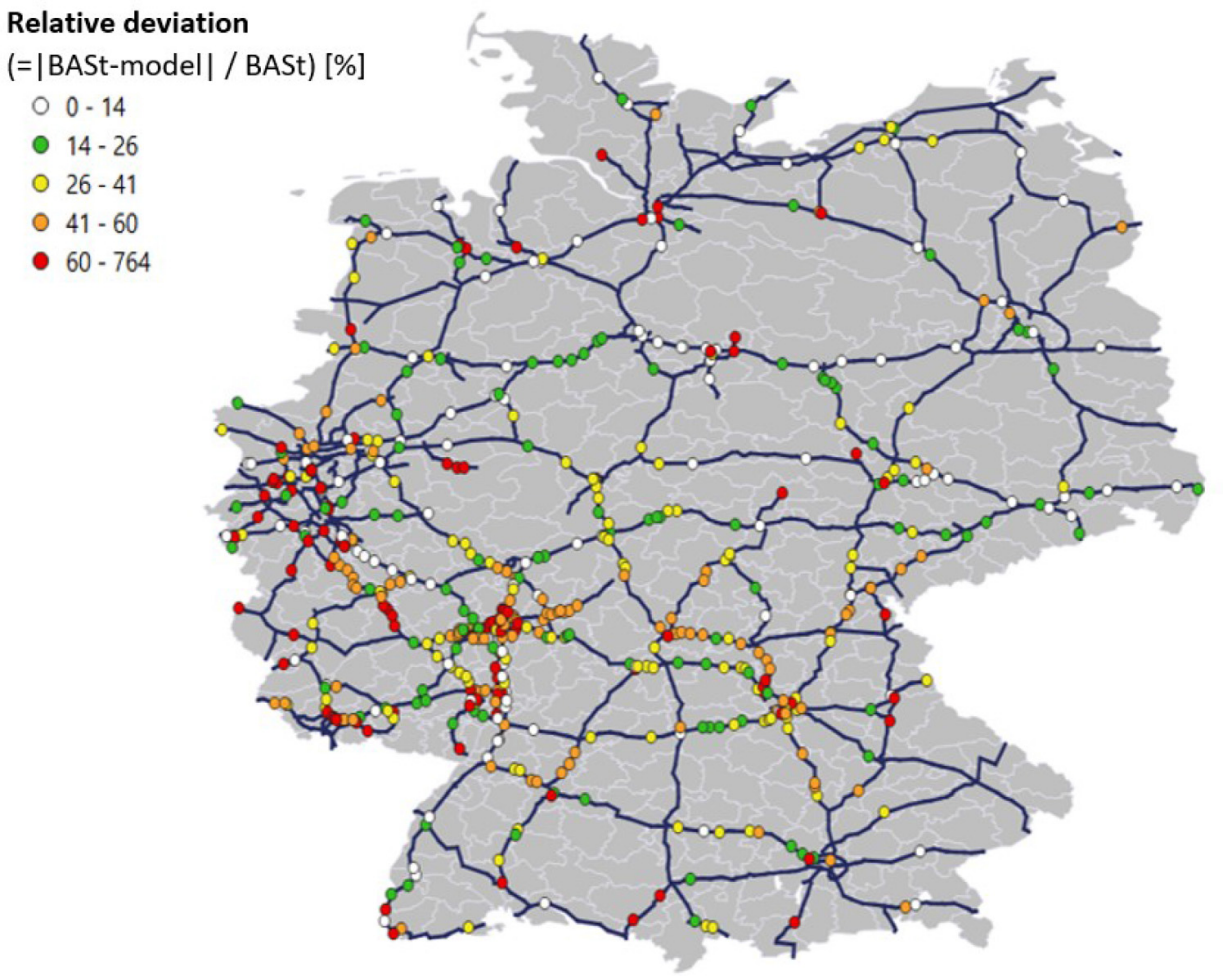
Relative deviation between traffic flows in model and BASt counting data [10].

In order to give an impression of the data quality of the developed dataset, Fig. 2 provides a comparison with data from the automatic traffic census in Germany [10] as an example. On long haul routes and between cities, the dataset reaches a high degree of consistency. The consistency in urban, densely populated areas is lower. Here, part of the traffic within a NUTS-3 region takes place on the E-road network, but it is not included in the modeled data. In addition, the simplifying assumptions described in Section 2 (Routing), for example the exclusive choice of the shortest route for two routes of nearly similar length, have a greater impact in densely populated areas with a dense transportation network. This should be taken into account when working on the presented dataset.

## 2 Experimental Design, Materials and Methods

The data used to develop the traffic model are based on the results of the European Transport policy Information System (ETIS) [1]. ETISplus 2010 represents an extension of its predecessor project, which ended in 2005, and to date provides one of the most comprehensive surveys of European transport. The origin-destination *Road Freight Matrix* within the ETISplus dataset [5] serves as the data basis for modeling transport flows for the dataset presented here. Numerous transport data tables from Eurostat, as well as national databases, were used within the ETISplus project to generate the origin-destination matrix (O-D matrix). The ETISplus dataset maps the transported goods volumes between the NUTS-3 regions of Europe.

### 2.1 Update of road freight transport volumes

The following describes how we first scaled up the ETISplus data to current numbers from 2019 and then projected to 2030. The O-D matrix of the ETISplus dataset is based on transport volume data collected by Eurostat [2]. To achieve the highest possible consistency, the scaling is also based on these data tables. For the representation of national transport flows, the two tables *road_go_na_rl3g* and *road_go_na_ru3g* are relevant. In the *road_go_na_rl3g* dataset, annual national transports are broken down by the respective NUTS-3 regions in which the goods were loaded. The transport volumes are given in 1,000 tons. The *road_go_na_ru3g* dataset shows the same transport volumes but breaks down the national transport quantities into the individual unloading regions. However, the data availability is quite incomplete - several years and even countries are not available mainly due to data confidentiality - and therefore the datasets cannot be directly adopted for the upscaling. Therefore, we calculate a country-specific growth factor based on aggregated national and international transport flows, which we use to adjust the ETISplus values from 2010 to 2019.

To calculate the national growth rate, the current value from 2019 is taken from the *road_go_na_tgtt* table in Eurostat [2]. The table contains the annual national transport volume of each country. These transport volumes are additionally supplemented by the annual road cabotage from the Eurostat table [2] *road_go_ca_hac*. Road cabotage is the transport of goods by a vehicle registered in one country, carried out in the national territory of another country. From the aggregated transport volumes, an average growth rate is calculated using formula (1) for each of the EU28 countries, England, Norway, and Switzerland, as data is only provided by Eurostat for these countries. The growth rate corresponds to the relative change in the transport volume of the individual countries compared to the previous value from the year 2010.

(1) $$\begin{array}{c} Average\;growth\;rate\;\bar{p}={\left(\frac{X_{n}}{X_{0}}\right)}^{\frac{1}{n}}-1\\ Here\;:\;{\bar{p}}_{country\;i}={\left(\frac{X_{i,\;\;2019}}{X_{i,2010}}\right)}^{\frac{1}{9}}-1\;\;\; \end{array}$$

${\bar{p}}_{country\;i}$Average growth rate in country i

$X_{i,\;\;2019}$Aggregated transport volume in country i in year 2019

$X_{i,2010}$Aggregated transport volume in country i in year 2010

The resulting country-specific growth rates are then applied to all national transportation flows in ETISplus at the NUTS-3 level using formula (2) to obtain updated values for 2019.

(2) $$\begin{array}{c} Scaled\;value\;X_{n}=\;{\left(1+\bar{p}\right)}^{n}*X_{0}\\ \;\;\;\;Here:\;X_{i,\;2019}=\;{\left(1+{\bar{p}}_{i}\right)}^{9}*X_{i,2010} \end{array}$$

To calculate the growth rates for the international transport flows, the growth rates of the exports of all EU28 countries, England, Norway, and Switzerland are considered separately. Since the average growth rates of exports (3.7%) and imports (3.64%) hardly differ from each other, the country-specific export growth factor is used to scale all international transport flows. Due to the large number of missing values, the growth rate can only be calculated for half of the countries from the export flows provided at NUTS-3 level (*road_go_ta_rl*). For those countries where the data set contains too many values that are not available in Eurostat [2], the aggregated exports from the table *road_go_ia_lgtt* are used.

To be able to analyse the charging infrastructure required in the future, the current traffic flows must be projected to the year 2030. Since no single growth value can be found in the literature - the European Commission quotes values between 26% and 40% in different publications [11], [12], [13] -, it is assumed that the countries will continue to grow between 2019 and 2030 with the same growth rates as between 2010 and 2019.

In addition to the EU28 countries, England, Norway, and Switzerland, the ETISplus dataset also includes other countries on the European continent that are not EU member states. Import and export volumes of these countries correspond to only 0.118% of the total ETISplus transport volume. Since no values are available in Eurostat for these countries, the average growth rate of 25% is assumed in the corresponding cases.

It should be mentioned, that this trend-based approach does not reflect sudden changes in trade patterns between regions.

The following description summarizes the procedure:

1. Calculate growth rates:
  a. Determine growth rate for national transport:
    i Annual national transport for 2019: *road_go_na_tgtt*
    ii. Annual national cabotage for 2019: *road_go_ca_hac*
    iii. Total national transport for 2010: *ETISplus*
    iv. FOR each country:
      Calculate average national growth rate: ${\frac{\left(national\;transport\;2019\;+\;national\;cabotage\;2019\right)}{total\;national\;transport\;2010}}^{\left(\frac{1}{9}\right)}-1$

  b. Determine growth rate for international transport:
    i. Annual international export for 2019:
      IF: NUTS-3-level contains valid data:
      USE: *road_go_ta_rl*
      ELSE: USE *road_go_ia_lgtt* (aggregated)
    ii. Total international export for 2010: *ETISplus*
    iii. FOR each country:
      Calculate international growth rate: ${\frac{international\;export\;2019\;}{international\;export\;\;2010}}^{\left(\frac{1}{9}\right)}-1$

2. Update ETISplus data:
  a. Scale data to 2019:
    FOR each country:
     IF Country is within EU28, England, Norway, and Switzerland
      i. Update the current national traffic flows in ETISplus using the average national growth rate: $\;{\left(1+{\bar{p}}_{i}\right)}^{9}*national\;traffic\;flow\;ETISplus$
      ii. Update the current international traffic flows in ETISplus using the average international growth rate: ${\left(1+{\bar{p}}_{i}\right)}^{9}*international\;traffic\;flow\;ETISplus\;$
   ELSE: Update current traffic flows using total average growth rate of all countries

b. Scale data to 2030:FOR each country:
  IF Country is within EU28, England, Norway, and Switzerland
  i. Update the national traffic flows calculated for 2019 using the average national growth rate: $\;{\left(1+{\bar{p}}_{i}\right)}^{9}*national\;traffic\;flow\;2019$
  ii. Update the current international traffic flows calculated for 2019 using the average international growth rate: ${\left(1+{\bar{p}}_{i}\right)}^{9}*international\;traffic\;flow\;2019\;$
 ELSE: Update current traffic flows using total average growth rate of all countries

### 2.2 Conversion from road freight volumes to number of trucks

Loading factors convert the freight volume into vehicles travelling. In 2010 according to the European Commission, the average loading factor for trucks was 13.6 tons. This value remained constant between 13 and 14 tons in subsequent years [3]. Based on the constant developments of the loading factor in the EU described above, an average value of the loading factors of 13.6 tons for the years 2010, 2019 and 2030 is assumed for the calculation of the transport flows.

The average loading factor in Eurostat refers to transports of loaded trucks and the route calculation refers to transported freight volumes, which is why empty runs are not considered in the model evaluation so far. The average percentage of empty runs within the EU-27 countries was 20% of heavy goods transport in 2018. This can explain a difference factor of 1.25 between the model and BASt data. The adjustment of the traffic flows in the model therefore takes place based on the missing empty runs. The modeled number of trucks for each section is scaled with the value of 1.25 determined from the EU average. This is a simplification, as the proportion of empty runs usually varies between different goods and on different routes.

The following description summarizes the procedure:

1. Calculate traffic flows:
  a. Calculate average loading factor: Eurostat data
  b. Determine number of trucks: $\#trucks=\;\frac{transported\;goods\;[t]}{average\;loading\;factor}$
2. Adjust values due to empty runs:

$\#trucks\_updated=\#trucks_{old}*average\;share\;of\;empty\;runs$

**Fig. 3:**
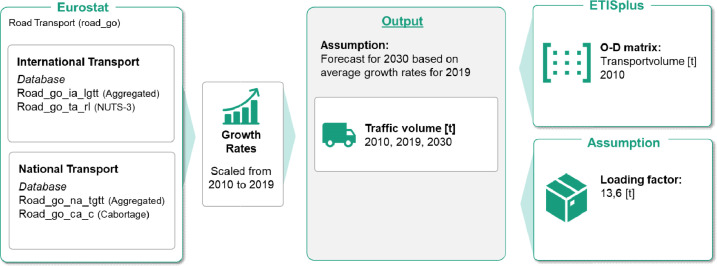
Graphical representation of the data processing.

Fig. 3 summarizes the data processing procedure.

### 2.3 Road network development

The highway network relevant for trucks was extracted from the ETISplus road network, which is part of the land networks [6], and manually updated with the current E-road network. The ETISplus table *Road Node* contains the relevant information to describe the nodes and the *Road Link* table to define the edges. Using all network elements in the ETISplus dataset results in a complex and disjointed road network with numerous edges. To focus on long-haul routes and to lower complexity, the network is reduced to road sections that are part of a highway or the international E-road network. Thus, only road sections that have the attribute M (highway), ME (highway and part of European road network), D (four-lane road), DE (four-lane road and part of European road network) or OE (side road and part of European road network) in the ETISplus dataset are used for modeling.

The resulting modeled network graph is neither complete nor fully connected, due to missing road segments, outdated classifications of roads and the general age of the ETISplus data. To ensure that all E-roads are part of the final graph, the connections of all European roads [14] are checked, and the missing edges are manually added. The lengths and thus weights of the supplemented edges are determined using the Google Maps API. Important ferry connections, which are needed to guarantee a coherent network, are also added manually. The length attributes of the ferry connections correspond to the opportunity costs, which are determined by the duration of the ferry trip and the speed of a truck. The speed of a truck on the highway is assumed to be 80 km/h.

### 2.4 Routing

To link the transport flows with the road network the corresponding transport routes need to be determined. First, every NUTS-3 region is assigned to a network node by calculating the shortest distance between the middle point of a region and all network nodes. These nodes define the start and ending points of each transport route. The Python library NetworkX [4] provides a variety of different methods for determining an optimal route within the modeled graph. For the determination of routes with minimum distances, Dijkstra's algorithm is used. For each O-D pair within the traffic flow matrix, an optimal route is computed in terms of edge and node paths using Dijkstra's algorithm.

This approach comes with some simplifications: (1) The algorithm always chooses the shortest route. (2) Each region is assigned to exactly one network node at which transport routes start and end. (3) If a transport process takes place exclusively within a NUTS-3 region, it cannot be mapped in the highway network.

In addition, all routes defined as regional traffic are excluded from the analysis, since the regional grid of NUTS-3 regions is not dense enough to map these transports in a meaningful way. Regional traffic includes all routes that do not have a network node within either the origin or destination region and are less than 50 km apart or directly adjacent.

After removing regional transports, the traffic volumes of the remaining routes are linked to the road network. In this process, the annual number of trucks for each network node and network edge is calculated using the optimal routes of Dijkstra's algorithm.

The following description summarizes the procedure:

1. Create E-road Network:
  a Create a graph representation of the European road system using python package *NetworkX*:
    i Use the table *Road Node* from ETISplus to define the nodes of the graph
    ii. Use the table *Road Link* from ETISplus to define the edges of the graph. Use the attribute *distance* as edge-weights
  b. Filter roads: only road sections that have the *RoadClass* attribute characteristics M, ME, D, DE or OE are used for modeling
  c. FOR each missing connections between nodes:
     Insert edge AND calculate distance with Google Maps API
  d. FOR main ferry routes:
     Insert edge AND calculate distance using opportunity costs
    distance=traveltime*80km/h

2. Calculate optimal routes:
  FOR each pair of NUTS-3 regions (origin-destination-pairs):
   Calculate optimal routes by finding edge-paths with minimal edge weights (distance) using Dijkstra's algorithm^2^

3. Calculate number of trucks for each road section:
  a. Delete regional traffic:
    i. Delete all intra-regional traffic flows with (origin region = destination region)
    ii. Delete all traffic flows (without a network node in origin region OR without a network node in destination region) AND (less than 50km apart OR adjacency regions)
  b. Assign aggregated truck flows to network edges:
    FOR each origin-destination pair:
     FOR each edge in the corresponding edge-path:
       Add traffic flow between origin-destination pair to total number of trucks on the edge

## CRediT authorship contribution statement

**Daniel Speth:** Conceptualization, Methodology, Software, Formal analysis, Data curation, Writing – original draft. **Verena Sauter:** Methodology, Software, Validation, Formal analysis, Investigation, Data curation, Writing – original draft, Visualization. **Patrick Plötz:** Conceptualization, Methodology, Writing – review & editing, Supervision, Project administration, Funding acquisition. **Tim Signer:** Methodology, Software, Validation, Formal analysis, Investigation.

## Declaration of Competing Interest

The authors declare that they have no known competing financial interests or personal relationships which have or could be perceived to have influenced the work reported in this article.

## Footnotes

1 The data set follows the division into NUTS regions (Nomenclature des unités territoriales statistiques) already used in the ETISplus project [1]. Here, level 0 describes countries, level 1 describes major socio-economic regions, level 2 basic regions for the application of regional policies, and level 3 small regions for specific diagnoses [8].

## Footnotes

2 shortest_node_path = networkx.shortest_path(E_road_network, origin, destination, weight = “distance”, method = “dijkstra”) Based on the shortest_node_path, Edge_path_E_road and all distances can be calculated using 02_NUTS-3-Regions, 03_network-nodes and 04_network-edges
